# Vitamin D Deficiency and *Toxoplasma* Infection

**Published:** 2019-06

**Authors:** Zohre FAKHRIEH KASHAN, Saeede SHOJAEE, Hossein KESHAVARZ, Mohsen ARBABI, Mahdi DELAVARI, Mahbobeh SALIMI

**Affiliations:** 1. Department of Medical Parasitology and Mycology, School of Public Health, Tehran University of Medical Sciences, Tehran, Iran; 2. Department of Medical Parasitology, School of Medicine, Kashan University of Medical Sciences, Kashan, Iran

## Dear Editor-in-Chief

Toxoplasmosis the zoonotic disease with worldwide distribution is life-threatening in congenital from and in immunocompromised patients (1). According to seroepidemiological studies, 18%–85% of infection is reported from different parts of Iran (2, 3). Both humoral and cellular immune systems are involved in *T. gondii* infection (4). In addition regulation role in calcium and phosphorus metabolism, vitamin D is an immune-modulator (5). On the other hand 1, 25-(OH) 2 D can act as anti-proliferative agent in mononu-clear cells (5) and reduce the production of IL2 and prevention of auto-immune diseases. Vitamin D deficiency is increasing in the world (6) and it is estimated 70% in Iranian people (7).

Based on this project, from the referred patients to Medical Laboratory in Kashan, Iran, 70 individuals checked for vitamin D were selected. People taking vitamin D supplements in the last 3 months and patients with chronic diseases were excluded from the study. Blood samples were conducted after 10 h of fasting. Vitamin D levels and anti-*Toxoplasma* IgG antibody were checked by commercial kit (EUROIMMUN, Germany) and ELISA method respectively. The mean age of participants was 40 yr, 72% were female and 28% were male. Average vitamin D levels were 9.9 ng/ml in deficient group and 67.23 ng/ml in normal group. Anti-*T. gondii* IgG antibody was positive in 17.14% of normal vitamin D group and 28.57% in vitamin D deficient group (*P*≤0/05 ((Tables 1, 2, 3). Statistical analysis was done by SPSS software (ver. 16, Chicago, IL, USA) and results expressed as mean ±SD. The significant differences of values were analyzed using Student’s t-test and one-way ANOVA test (*P*≤0.05).

**Table 1:** Average concentration of vitamin D (ng/ml) and percent of *Toxoplasma* seropositivity in groups A, B

***No.***	***Type***	***Vitamin D average concentration(ng/ml)***	***Toxoplasma seropositivity (%)***
Group A (35)	Vitamin D deficient	9.9	28.57
Group B (35)	Normal vitamin D	67.23	17.14

**Table 2:** Average concentration of vitamin D (ng/ml) and percent of *Toxoplasma* seropositivity in age groups of group A

***Age (yr)***	***No.***	***Vitamin D average concentration (ng/ml)***	***Toxoplasma seropositivity No. (%)***
0–10	1	16.26	-
11–20	3	4.6	-
21–30	7	7.77	1(2.85)
31–40	14	7.38	5 (14.28)
41–50	5	13.6	3 (8.58)
>50	6	10.35	1 (2.85)
Total	35	9.9	10 (28.56)

One-Way ANOVA test (*P*<0.05)

**Table 3:** Average concentration of vitamin D (ng/ml) and percent of *Toxoplasma* seropositivity in age groups of group B

***Age (yr)***	***No.***	***Vitamin D average concentration (ng/ml)***	***Toxoplasma seropositivity No.(%)***
0–10	4	56.62	1 (2.85)
11–20	-	-	-
21–30	5	49.23	1(2.85)
31–40	3	60.4	-
41–50	12	55.58	3 (8.5)
>50	11	47.11	1 (2.85)
Total	35	67.23	6 (17.14)

One-Way ANOVA test (*P*<0.05)

There was widespread high prevalence of vitamin D deficiency in populations (6, 7). On the other hand, *T. gondii* infection is one of the most prevalent infectious diseases worldwide.

Regarding the results of this study, the difference in prevalence of *T. gondii* infection in two groups of vitamin D sufficient and deficient individuals was noticeable and *Toxoplasma* infection was associated with vitamin D deficiency. More studies are suggested for in vivo and in vitro interpretation of vitamin D and parasitic infections.
